# Effects of Boric Acid on Laminated Composites: An Experimental Study

**DOI:** 10.3390/polym16152133

**Published:** 2024-07-26

**Authors:** Gurbet Örçen, Duygu Bayram

**Affiliations:** 1 Department of Mechanical Engineering, Dicle University, Diyarbakır 21280, Turkey; 2 Institute of Science, Dicle University, Diyarbakır 21280, Turkey; dyg_bayram@hotmail.com

**Keywords:** boric acid, mechanic properties, thermal properties, failure analysis, nanocomposite

## Abstract

In this study, the effect of boric acid (H_3_BO_3_) on fiber-reinforced layered composites was investigated. Glass fiber-reinforced epoxy composites were used, and the effects of boric acid on thermal and mechanical properties were investigated. For this purpose, composite plates were manufactured by adding boric acid (BA) to the epoxy in different ratios (0, 0.5, 1, and 1.5% by weight). Tensile tests, compression tests, and shear tests were performed to determine the mechanical properties of these plates, and DSC, TGA, and DMA analyses were performed to determine their thermal properties. SEM and EDS analyses were performed on the specimens to examine their morphologies. Furthermore, examinations were conducted on how BA affected the specimens’ failure behavior. In the study, it was found that, except for the compressive strength, the mechanical properties were improved by the added BA. The highest tensile strength, shear strength, modulus of elasticity, shear modulus, and Poisson’s ratio were obtained from 0.5% BA-added specimens and were 24.78%, 8.75%, 25.13%, 11.24%, and 12.5% higher than the values obtained from 0% BA-added specimens, respectively. The highest loss and storage modulus were obtained from 0% and 0.5% BA-added specimens, respectively. The specimens’ glass transition temperatures were decreased by the addition of BA; the specimen with a 1% addition of BA had the lowest value. Furthermore, interlayer delamination and fiber/matrix failure were observed in all BA-added specimens.

## 1. Introduction

Epoxy resins are widely used for coatings, electronic materials, adhesives, and fiber-reinforced composites due to their outstanding mechanical properties, high adhesion strength, good heat resistance, and high electrical resistance [1]. However, the flaws of epoxy, such as its flammability and inherently brittle nature, have limited its application [2]. In this sense, different types of nanoparticles are applied to enhance epoxy composites’ mechanical and thermal characteristics. Nanoparticles are very promising for improving the properties of composites [3]. These nanoparticles, which contain boron and its compounds, have created an important field of application due to their use in resin or reinforced material and their improvement of the properties of the composite material. Boron compounds [4], such as boric acid, are well-known fire retardants [3,4] and have an active insulating effect [3].

In the literature, there are many studies on the improvement of mechanical properties in metal matrix composites [5,6,7,8,9,10,11,12,13], among which boron and its compounds are used. There are also studies in the literature on the use of boron carbide (B_4_C), boron nitride (BN), and boron in polymer composites. It has been reported that the wear resistance [14], tensile [15], bending [15,16], hardness, and fracture toughness [16] properties of the composite are improved by using boron carbide in polymer composites. It was stated that the elasticity modulus, stiffness [17], mechanical, and tribological properties [18,19] of the composite were improved by the use of boron nitride, but the strength decreased [20] as the BN ratio increased. In addition, the effect of boron use on the failure behavior [21] and thermal stability [22] of composites has been emphasized in the literature.

Only a limited number of studies [23,24,25,26,27,28,29,30,31,32,33,34,35,36,37,38,39,40] have been published in the literature on the usage of BA and other nanoparticles with BA in epoxy composites. Tuisov et al. [23] proposed a method for modifying epoxy binder by adding a modifying additive, boron polymer, to increase its strength. In their study, they showed that their proposed method was effective. Karua and Arifüzzaman [24] produced slightly expanded perlite/sodium silicate composite foams with boric acid (BA) in amounts ranging from 0 to 2.88% by weight. Then they investigated the compression behavior of these composite foams. They found that the compressive strength and compressive modulus of the foams increased by 0.74% with the weight of boric acid content. Pehlivanlı [25] investigated the effects of boric acid additives on the microstructure formation and thermal properties of polypropylene. Composites were produced by adding particulate boric acid to polypropylene with the help of an injection molding machine. The author stated that the tensile stress and strain of the composite increased with the increase in the mass ratios of BA. In their investigation, Bağcı and İmrek [26] added BA at a rate of 15% of the resin as filler material to pure glass fiber-reinforced epoxy resin composites. They investigated the structure and erosion wear behavior of the material at different impact velocities at 30, 60, and 90° impact angles in 0 and 45° fiber directions with an aluminum erodent with particle sizes of E200 and 400 mm. They stated that the addition of boric acid filler material in GF/EP composites reduces the hardness, tensile strength, modulus of elasticity, and density values. Polat and Kaynak [27] investigated the feasibility of using boron oxide (BO) and BA to enhance the flame retardancy of organophosphorus compounds in polyamide-6 (PA) and its 15 wt% short (glass fiber) reinforced composite. Jian et al. [28] reported improvements in thermal and mechanical properties in their study on nanoboronium-reinforced polyimide composites with greatly improved thermal and mechanical properties through in situ thermal conversion of BA. The effects of surface-modified BA on the chemical and physical characteristics of composites based on polyethylene were examined by Uddin et al. [29]. They reported that the thermal degradation activation energy obtained from BA-added specimens was higher than that obtained from non-BA-added specimens, but the elongation values were lower. Visakh et al. [30,31] observed that the decomposition temperatures of composites at different stages increased with the addition of BA and that thermal stability increased as the amount of BA increased [30]. They prepared epoxy composites filled with BA and natural zeolite at different ratios (1, 5, and 10 wt%) [31]. They reported that the composite filled with 10 wt% BA showed the best improvement in the thermal properties of the composites due to the endothermic degradation of BA on heating [31]. Nazarenko et al. [32,33,34] prepared ED-20 as epoxy resin, polyethylene polyamine as hardener, aluminum nano powder and BA as flame retardant fillers [32], 0.5% carbon nanotubes (MWCNT), BA and sodium bicarbonate (10, 15%) [33], and zeolite particles with 15% BA [34]. They noted that the best thermal stability could be achieved by using a combination of boric acid [32,33] and multi-walled carbon nanotubes [33]. However, they found that the addition of 0.5% MWCNTs by weight to the epoxy matrix did not improve the thermal stability of the composite [33]. They noted that the addition of all fillers improves the thermal stability and mechanical properties of epoxy composites [32]. They also stated that the combination of 5% zeolite and 10% BA by weight significantly increased the middle point temperature in tortoises [34]. Zhu et al. [35] have worked to produce an advanced thermal insulation material with excellent performance. For this purpose, they fabricated an anisotropic composite aerogel based on cellulose nanofibers (CNF), calcium alginate (CA), and boric acid (BA). In their study, the authors stated that CA and BA provide the aerogel with good mechanical strength and excellent flame-retardant properties. Hamciuc et al. [36] added a phosphorus-containing DOPO (9,10-dihydro-oxa-10-phosphophenanthrene-10-oxide) derivative (PFR) and BA to the resin to increase the flame resistance of epoxy resin. They studied the effect of PFR containing BA and phosphorus on reducing the flammability of epoxy resin. The authors reported that specimens containing BA were beneficial in terms of higher char yield and improved flame retardancy. Avcı et al. [37] investigated how boric acid–borax mixtures (1:1) and zinc borate fillers affect the thermal and mechanical properties of poly(lactic acid) (PLA). They added three different boron fillers to the PLA matrix at four different concentrations of 3%, 5%, 8%, and 12% by weight. In their study, they reported that, although the tensile and flexural strengths of PLA decreased, the flexural and Young’s moduli increased by approximately 20% and 22%, respectively, with increasing boron filler ratios. Rudawska et al. [38,39] compared selected mechanical properties of unmodified epoxy resin-based epoxy compounds with those containing antiseptics as a modifying agent [38]. They also investigated the effect of BA by adding three different amounts (0.5 g, 1.0 g, and 1.5 g) of BA powder to epoxy resin [38,39] to obtain a composite material with improved properties and performance [39]. They carried out their experimental work on 12 epoxy composites made of bisphenol A (BPA)-based resin [38,39] as the matrix of epoxy composites [38]. The compressive strength of cured, modified, and unmodified epoxy compounds was investigated [38]. They also evaluated the effect of BA addition and the type of curing agent used on the mechanical properties of the epoxy composites they obtained [39]. In the study, it was stated that compressive strengths decreased and increased according to polyamide, amine curing agent rates, and BA additive rates [38,39]. Yoğurtçu et al. [40] investigated the effect of BA on the morphological, mechanical, barrier, and optical properties of thermoplastic starch. In their study, they used BA at 0.5%, 1%, 2%, 4%, and 8% by weight. In their study, the authors stated that, with the addition of 0.5% BA, water vapor permeability, water solubility, and swelling properties decreased, but mechanical properties were positively affected.

The aim of this study is to investigate the effect of BA on the mechanical and thermal properties of fiber-reinforced epoxy composites. Therefore, BA additives of 0%, 0.5%, 1%, and 1.5% by weight were used in glass fiber-reinforced epoxy composites to produce layered plates. The thermal and mechanical properties of glass fiber-reinforced epoxy composite plates used in this study have been previously investigated using the same ratios of nanoclay [41]. In the literature review, it was observed that there are very few studies on the use of BA alone in the production of polymer-based composite plates. In order to contribute to the literature and to investigate the effect of BA, DSC, TGA, and DMA analyses and mechanical tests were performed. In addition, the distributions of BA were analyzed using SEM and EDS analysis. At the same time, the failure modes obtained after the tensile test were evaluated. The data obtained were presented and compared in tables and graphs. The results obtained will contribute to the use and design of nanocomposites in application areas.

## 2. Materials and Methods

**Figure 1:**
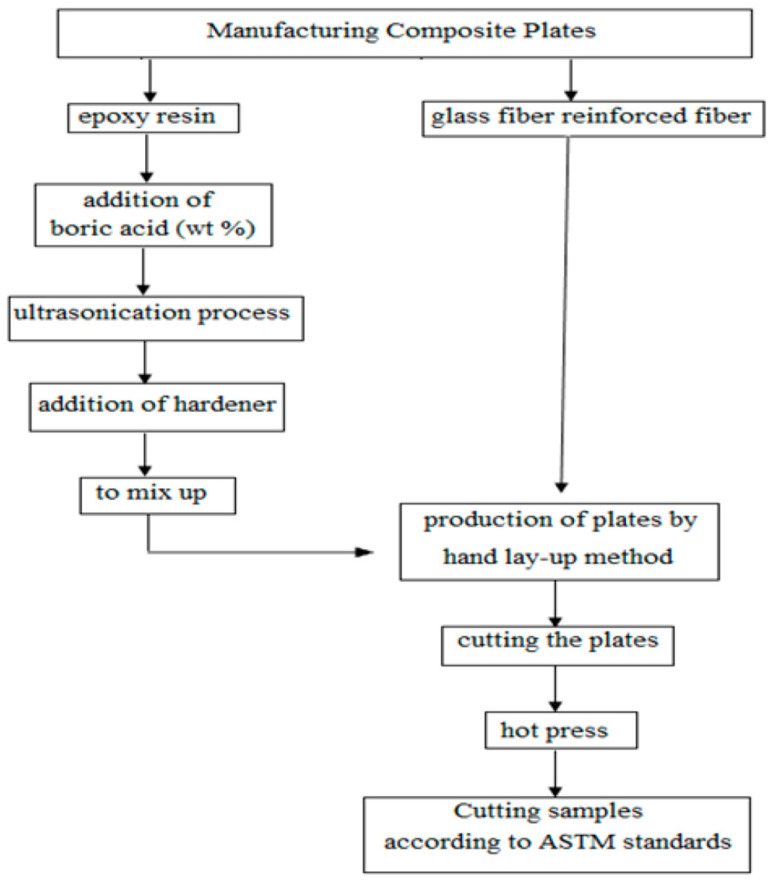
Production processes.

**Figure 2:**
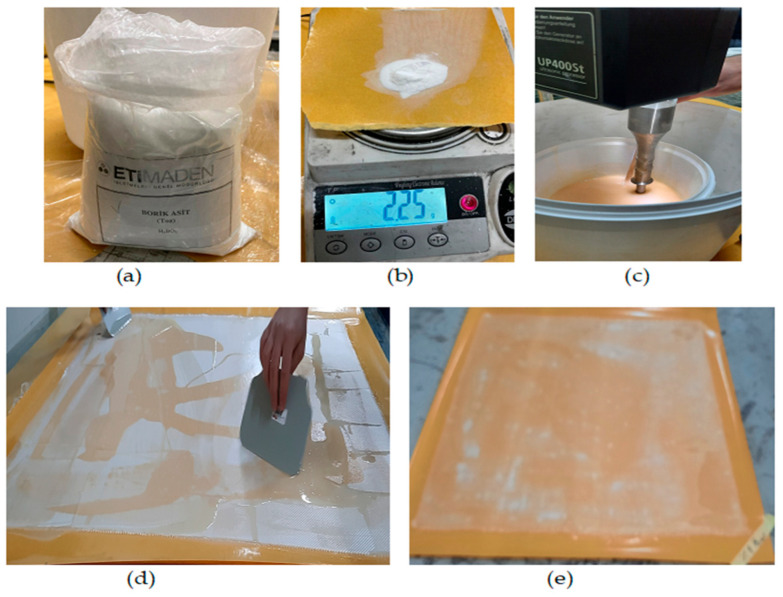
Procedures for manufacturing hand lay-up (**a**); BA (**b**); precision balance (**c**); mixer (**d**); applied to the fabric by hand laying method (**e**); resin impregnated fabric.

The production scheme for obtaining BA-added composite plates and specimens is given in Figure 1. An epoxy resin (1.15 g/cm^3^, FRES 21, Fibermak, İzmir, Turkey) with E-Glass woven fibers (300 gr/m^2^-Twill) was used in the study. The powder BA (H_3_BO_3_) with a molecular weight of 61.83 g/mol and a specific weight of 1.51 g/cm^3^ (20 °C) was obtained from the General Directorate of Eti-Maden Company (Ankara, Turkey) (Figure 2a). All productions were carried out in Fibermak company.

The amount of BA was weighed on a scale with a precision of 0.01 g (Figure 2b) and added to the epoxy resin. The BA-epoxy mixture was first mechanically stirred with a wooden stick for about 1 min. The epoxy resin was then mixed using an ultrasonic mixer (Hielscher UP400S, Hielscher Ultrasonics GmbH, Germany) (Figure 2c). The device, which works by generating sound waves, was applied to the mixture for at least 60 min. The temperature of the mixture was continuously measured with a digital thermometer to ensure that it did not exceed 50 °C. At the end of the mixing time, a hardener (FHARD 22, Fibermak, Izmir, Turkey) was added to the mixture, and the mixture was mixed for another 10 min.

These processes were carried out separately and at room temperature for all BA ratios. For the production of composite plates with eight layers and a thickness of 2.5 mm, this mixture was applied to the glass fiber fabric by the hand lay-up method (Figure 2d,e). 

**Figure 3:**
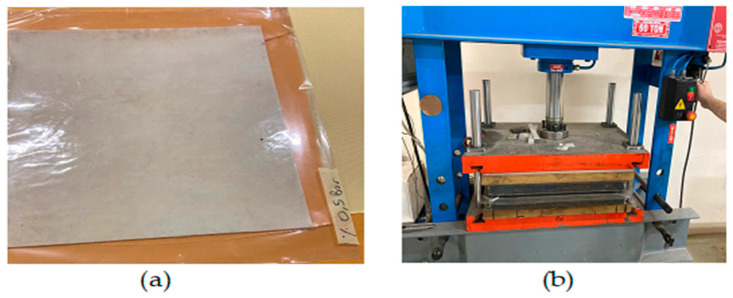
Production of BA-added glass fiber-reinforced composite plate: (**a**) wrapping in fireproof film; (**b**) placement in a hydraulic press.

This processing was carried out by experts who have been doing this work continuously. After processing, the fabrics were left to cure at room temperature. These semi-finished fabrics are stacked in eight layers and coated with a fireproof film (Figure 3a). They were then placed in the hydraulic press (Figure 3b). Initially, a pressure of 10 bars was applied. Then, the temperature was raised to 125 °C and kept at this temperature for one hour. At the end of the time, the heater was turned off and allowed to come to room temperature.

For the experimental procedures, specimens were cut by a CNC machine in accordance with ASTM 3039-17 [42], ASTM D 6641-16 [43], and ASTM D 7078 [44] for tensile tests, compression tests, and shear tests, respectively.

For every test, five specimens were prepared, and the tests were conducted at room temperature. In the tensile test specimens, the same composite material was glued to the head of the specimens with adhesive to prevent stress concentration. A device of the type Instron 8801 with a 100 kN capacity was used for the testing. The specimens were strain-gauged to determine the shear modulus, compressive stress, modulus of elasticity, and Poisson’s ratio.

Using the Vickers test method, the specimens’ hardness was determined. Five tests were performed at a load of 0.3 kg with a dwell time of 10 s and then averaged.

### 2.1. DMA Analysis

This analysis was performed to investigate the storage modulus (E’), loss modulus values (E″), and tan δ–temperature curves of the specimens. On the TA Instruments Q800 (Ege University, Izmir, Turkey), the specimens performed dynamic mechanical analysis (DMA). The ASTM D7028-07 standard [45] was followed when preparing the specimen dimensions. The dual cantilever mode was selected as the test method. The test was conducted using the following parameters: temperature range, room temperature to 150 °C; frequency, 1 hz; force, 1 N; amplitude, 15 µm; heating rate, 5 °C/min. 

### 2.2. DSC Analysis

Glass transition temperatures (Tg) of the specimens were determined using Differential Scanning Calorimetry (DSC) analysis on a Schimadzu DSC-60 (Dicle University, Diyarbakır, Turkey) type apparatus at a temperature increase rate of 10 °C/min up to 300 °C.

### 2.3. TGA Analysis

Thermogravimetric analysis (TGA) measures the change in weight of a specimen by temperature change, which can indicate the degradation or decomposition of the specimen [46]. A DTG-60H Shimadzu- brand device (Dicle University, Diyarbakır, Turkey) was used to perform the TGA. For the TGA analysis, the specimens were heated at a rate of 10 °C per minute from 23 °C to 800 °C.

### 2.4. SEM and EDS Analysis

The specimens were subjected to scanning electron microscopy (SEM) and energy dispersive spectrograph (EDS) analysis using the JEOL JSM-5600 device (Munzur University, Tunceli, Turkey). 

## 3. Results and Discussion

### 3.1. Mechanical Properties

**Figure 4:**
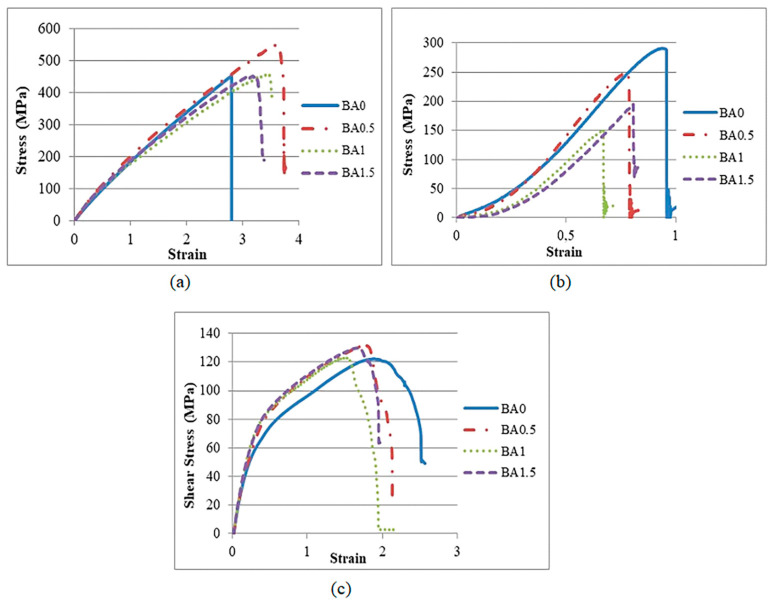
Comparison of the graphs obtained: (**a**) graphs of tensile tests; (**b**) graphs of compression tests; (**c**) graphs of shear tests.

Glass fiber-reinforced composites containing BA at four different ratios (0%, 0.5%, 1%, and 1.5%) performed tensile, compression, and shear testing. Comparative stress–strain graphs obtained from the experiments are given in Figure 4. The results and standard deviation values obtained at the same time are given in Table 1. In the tensile test results, the highest value was obtained from the BA0.5 specimen (Figure 4a). Similar tensile strength values were obtained from the specimens with BA1 and BA1.5 ratios. The value obtained from specimen BA0.5 increased by 24.78%, 20.58%, and 20.71% compared to the values obtained from specimens BA0, BA1, and BA1.5, respectively (Figure 4a, Table 1). 

In the compression test, the highest result was obtained from the BA0 specimen (Figure 4b). This demonstrates that compression strength is negatively impacted by BA. In comparison to the values obtained from the BA0.5, BA1, and BA1.5 specimens, respectively, the compression strength value obtained from BA0 was 15.69%, 48.13%, and 33.26% greater (Figure 4b, Table 1). The reduced compressive strength is thought to be a result of the weak adhesion between the layers.

The comparatively presented stress–strain graphs from the shear test are shown in Figure 4c. From the BA0.5 specimen, the highest value was found. The value obtained from the BA0.5 specimen increased by 8.75%, 4.89%, and 2.63% compared to the values obtained from the B0, BA1, and BA1.5 specimens, respectively (Table 1). 

The modulus of elasticity and shear modulus values increased with increasing BA ratios. Maximum modulus of elasticity and shear modulus values were obtained from the BA0.5 specimen. The modulus of elasticity obtained from BA0.5-, BA1-, and BA1.5-added specimens increased by 25.13%, 20.16%, and 14.64%, respectively, compared to BA0 specimens (Table 1). The shear modulus values obtained from BA0.5, BA1, and BA1.5 specimens increased by 11.24%, 3.04%, and 7.13%, respectively, compared to BA0 specimens (Table 1). 

Compared to the value obtained from the BA0 specimen, Poisson’s ratios increased as the BA ratio increased. The highest values were obtained from the BA0.5 and BA1.5 specimens (Table 1). 

From the specimen with a BA1.5 ratio, the highest hardness value was obtained. The hardness value determined from the BA0 specimen was found to be 13.55%, 22.58%, and 70.65% lower than the specimens with BA0.5, BA1, and BA1.5 ratios, respectively (Table 1). 

It was found that there are few studies on the use of BA in the production of fiber-reinforced epoxy composites or epoxy resins. In addition, BA has been used both in different ratios and in combination with different materials in the literature. The influence of an additive on the mechanical properties of an epoxy compound depends on many factors, starting with the type of matrix resin [39], and these variables can affect the strength parameters [38]. Therefore, the effect of BA on mechanical properties varies. Boric acid is a good crosslinking agent [35,40], and the tensile strength increases as crosslinking leads to a strong intermolecular interaction. However, tensile strength tends to decrease with the excessive addition of boric acid [40]. Bağcı and İmrek [26] reported a decrease in the tensile strength of the specimens to which 15% BA was added. Polat and Kaynak [27], on the other hand, stated that both the tensile strength and modulus of elasticity of the specimens with a 10% BA addition decreased. Avcı et al. [37] found that although the tensile strength decreased with increasing ratios, Young’s modulus increased. However, Pehlivanlı [25] stated that tensile strength increased with increasing rates (0.5%, 1.5%, and 2.5% BA-added). 

In this study, according to the tensile strengths obtained from the BA0 specimen, the tensile strength of the specimens increased with increasing BA ratios. Increases in modulus of elasticity, shear modulus, shear strength, and Poisson’s ratio were also obtained with increasing rates. However, the highest increase was obtained from the specimens at BA0.5. The decrease in BA1 and BA1.5 ratios is thought to be due to the incompatibility of epoxy and BA with the interface [25] and matrix [37]. Increasing wettability between fiber and polymer is a fundamental approach to enhancing interface bonding [46]. Insufficient wetting ability of the polymer matrix for fillers leads to poor filler–matrix adhesion. Furthermore, there may be boron filler agglomeration in a composite structure, resulting in increased stress at the matrix–filler interface. This can lead to a decrease in strength [25]. 

With the increase in BA ratios, the hardness values obtained from the specimens have also increased. Tasgin [13] noted that when used with Sn-Sb-Cu alloys with 10% BA, Avcı et al. [37] noted that the boron compounds added to PLA increased the hardness of the composite. However, when Bağcı and Imrek [26] used 15% BA in epoxy composites and Nazarenko [33] used 10–15% BA, they found that the hardness values obtained from the specimens decreased.

In this study, very good results were obtained on all mechanical parameters when BA was applied alone and in increasing ratios, while it had a negative effect on compressive strength. Rudaws et al. [39] reported that the compressive strength of boric acid/epoxy composites cured with amine and polyamide curing agents decreased with increasing boric acid content. Therefore, they stated that the addition of boric acid has a limited and negative effect on compressive strength [39]. 

Except for hardness, the values obtained from BA0.5 specimens were higher than the values obtained from BA1 and BA1.5 specimens.

### 3.2. Findings of the DMA Analysis

**Figure 5:**
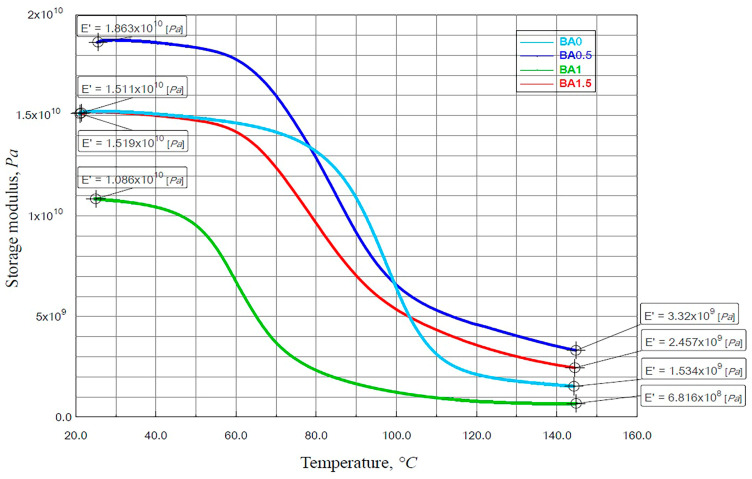
The storage modulus–temperature graph of specimens.

The storage modulus (E′) is an indicator of the modulus of composite materials, while the loss modulus (E″) is the energy lost due to the friction of polymer chain movement, and tan δ is very sensitive to structural transformation [47]. Therefore, DMA analysis was performed to investigate the effect of BA on storage modulus (E′), loss modulus (E″), and tan δ values. Figure 5 displays the specimens’ storage modulus–temperature graph. 

The hardness of the material was evaluated by the E′ [47]. The highest E′ was obtained at 18,630 MPa from the specimen with BA0.5 (Figure 5). Maximum tensile strength and modulus of elasticity values were obtained from the BA0.5 specimen (Table 1). In this sense, the results are consistent with each other. The specimens with the BA0, BA1, and BA1.5 ratios obtained values of 15,190 MPa [41], 10,860 MPa, and 15,110 MPa, respectively. For every specimen, the E′ value decreased as the temperature increased. At roughly 50 °C, a drop was seen for all specimens due to the glass transition area. The rubber plateau zone is the area where the E’ stabilizes at a constant width following the crossing of the glass transition temperature [19]. 

The specimen BA1 has the fastest transition to the plateau region, as shown in Figure 5. In this region, 1534 MPa, 3320 MPa, 681.6 MPa, and 2457 MPa were obtained in BA0, BA0.5, BA1, and BA1.5 specimens, respectively. While the specimens with BA0 and BA1.5 ratios initially had close values, the difference between them became apparent due to the movement of the polymer chains in the rubber region. 

**Figure 6:**
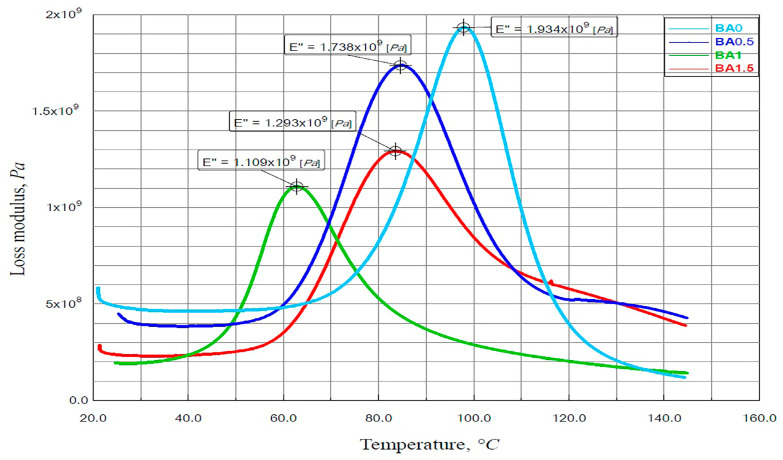
The loss modulus–temperature graph of specimens.

The loss modulus–temperature variation graph obtained from the specimens is given in Figure 6. The maximum point of the E″ represents the temperature at which the polymer chains undergo the maximum change in mobility. According to the experimental results, the highest loss occurred in specimen BA0. BA0, BA0.5, BA1, and BA1.5 ratios were obtained as 1934 MPa [41], 1738 MPa, 1109 MPa, and 1293 MPa, respectively.

**Figure 7:**
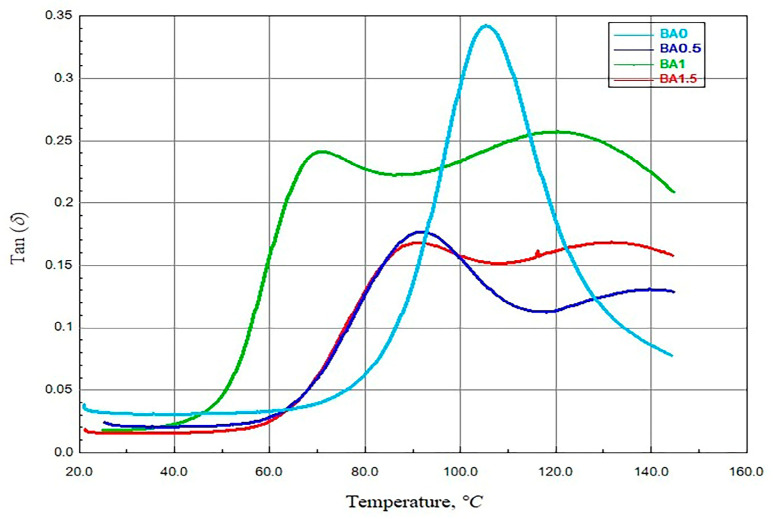
The tan δ -temperature graph of specimens.

Figure 7 shows the temperature-dependent tan δ graphs that have been obtained from the specimens. In the experiment, the tan value of all BA-added specimens was lower than the value obtained from the BA0 specimen. The tand value for BA0 was obtained as 0.340 [41]. BA0.5, BA1, and BA1.5 for specimens 0.1767, 0.241, and 0.1682 were obtained, respectively. The tan δ technique was used to determine the specimens’ glass transition temperatures. As a result, 104.62 °C [41], 91.839 °C, 70.949 °C, and 91.293 °C were found to be Tg of the BA0, BA0.5, BA1, and BA1.5 specimens, respectively. It is seen that Tg, which is affected by the interaction mechanism in the interfacial area and nanolayer formation in the polymer [47], decreases with the inclusion of BA in the epoxy matrix.

### 3.3. Findings of the TGA Analysis

**Figure 8:**
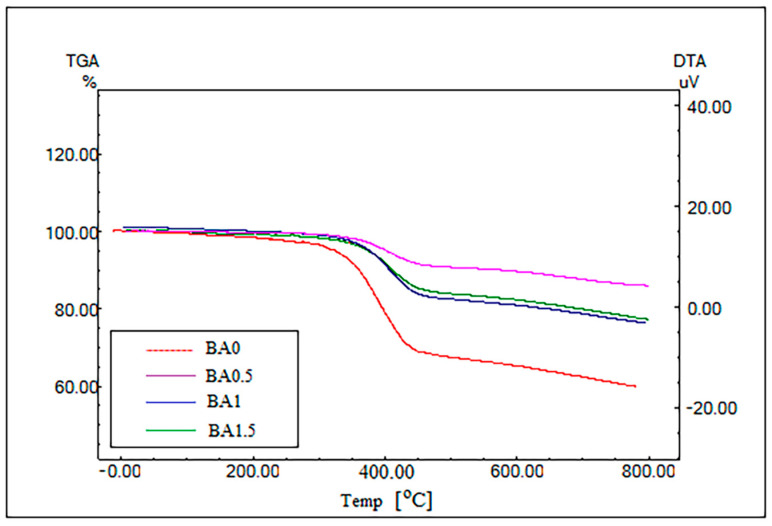
Comparison of the specimens’ TGA graphs.

The obtained TGA graphs are given in Figure 8. In the analysis, the thermal decomposition processes of the specimens took place in several stages.

In all specimens, the first decomposition temperatures were approximately in the range of 50–280 °C. Then, the highest loss occurred in the range of approximately 300–490 °C. Loss rates in this range are given in Table 2. The residual mass loss is observed in the range of approximately 500–800 °C. As seen in Figure 8, we can say that thermal stability increases with an increasing BA ratio. It is seen that the thermal degradation behavior of the specimens with BA1 and BA1.5 ratios is similar. This similar behavior is also seen in the similarity in loss rates (Table 2).

According to Visakh et al. [30], the addition of BA increased the thermal stability. In this study, the BA0.5 specimen showed the best result in thermal degradation. This result shows the effectiveness of using BA as an additive in epoxy resin to reduce flammability.

### 3.4. Results of DSC Analysis

**Figure 9:**
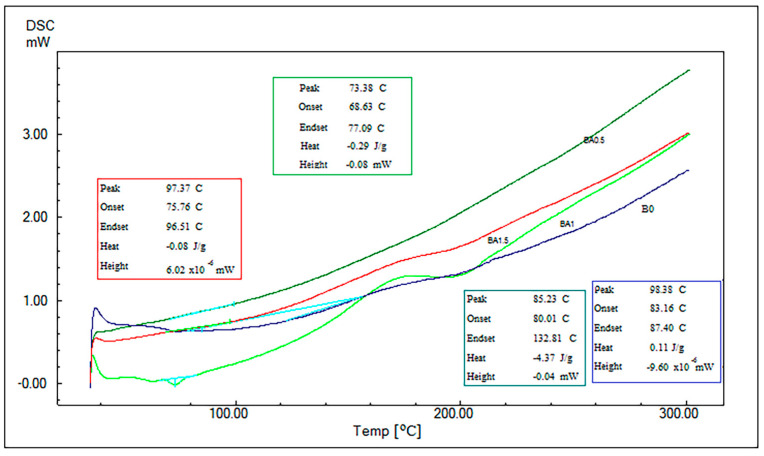
Comparative DSC graph.

Figure 9 presents a comparative analysis of the DSC graphs produced from the specimens. The Tg temperatures of BA0, BA0.5, BA1, and BA1.5 specimens were determined to be 98.38 °C [41], 85.23 °C, 73.38 °C and 97.37 °C, respectively. 

In the analysis, it was found that the addition of BA decreased the Tg temperatures compared to the value obtained from the B0 specimen. At 0.5 and 1% of added BA, this decrease was found to be higher, but this decrease decreased at 1.5%.

**Figure 10:**
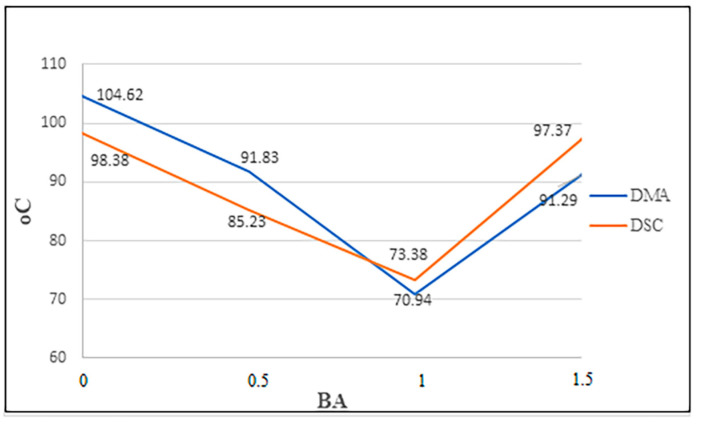
Comparison of Tg temperatures.

The results of the Tg temperatures obtained from the DMA analysis and the DSC analysis are compared in Figure 10. It is seen that the values obtained by both analyses are close to each other, and, in this sense, the data are compatible with each other.

Nazarenko et al. [32] reported that the Tg temperature decreased when 10% BA was used compared to the pure specimen. Avci et al. [37] found that the Tg temperature in PLA decreased gradually as the boric acid/borax ratio increased from 3% to 12%. Nazarenko et al. [33] reported that Tg increased when 10% and 15% BA were used compared to the pure specimen. However, depending on the kind of epoxy used and the BA ratio, these increases and decreases can differ. In this investigation, the DMA and DSC analyses resulted in the lowest values with the BA1 added; with the BA1.5 added, this value increased (Figure 10). If the BA ratio is increased further, its relationship with the Tg temperature can be investigated by other studies.

### 3.5. SEM and EDS Images

**Figure 11:**
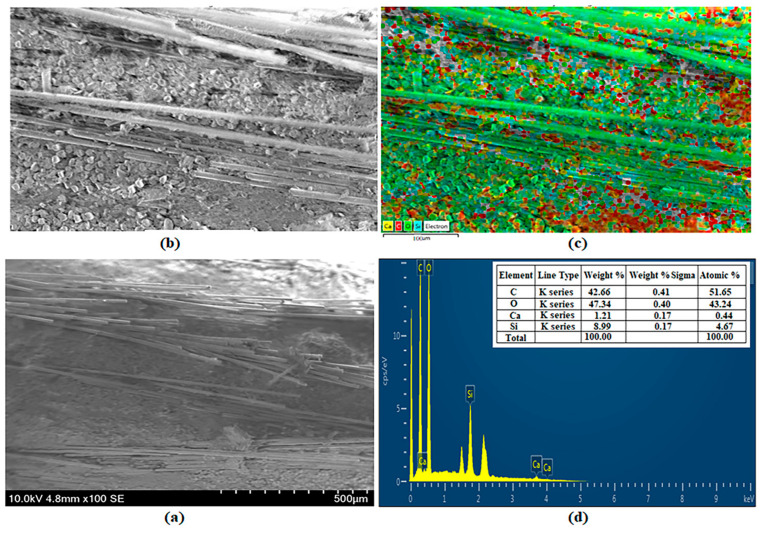
Analysis of specimen BA0: (**a**) SEM, (**b**) EDS [41], (**c**) Mapping [41], and (**d**) EDS [41].

**Figure 12:**
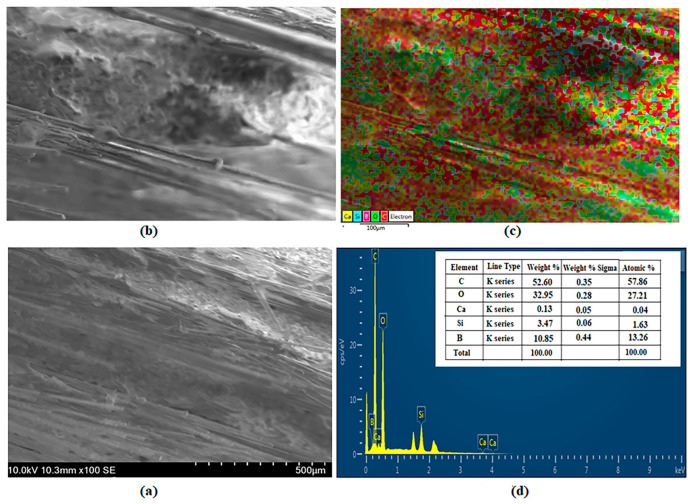
Analysis of specimen BA0.5: (**a**) SEM, (**b**) EDS, (**c**) Mapping, and (**d**) EDS.

**Figure 13:**
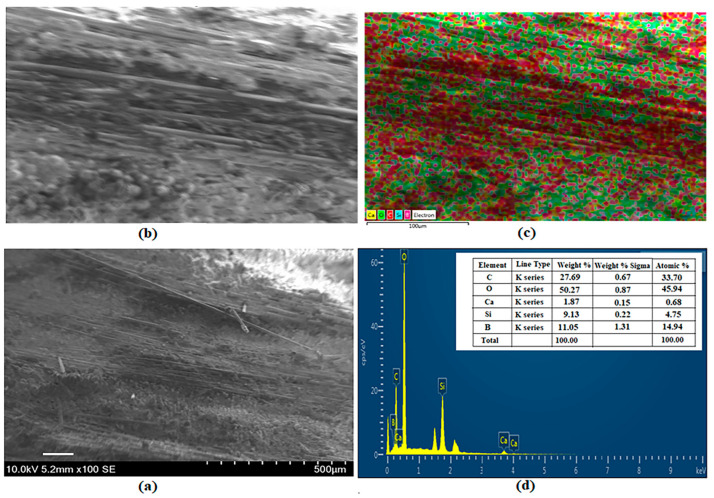
Analysis of specimen BA1: (**a**) SEM, (**b**) EDS, (**c**) Mapping, and (**d**) EDS.

**Figure 14:**
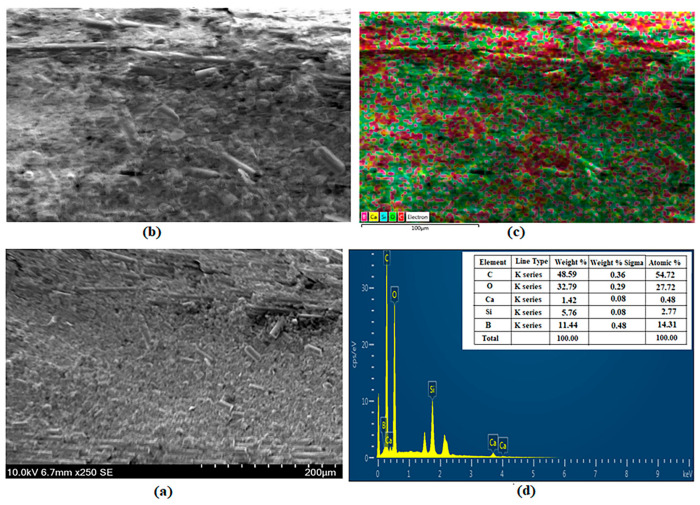
Analysis of specimen BA1.5: (**a**) SEM, (**b**) EDS, (**c**) Mapping, and (**d**) EDS.

SEM, EDS and mapping analysis, and EDS peak values of BA-added specimens are shown in Figure 11, Figure 12, Figure 13 and Figure 14. Figure 11 shows the analysis results for BA0. Ca (yellow), C (red), O (green), and Si (blue) are observed as 1.21%, 42.46%, 47.34%, and 8.99% by weight, respectively [41] (Figure 11c,d).

The analysis results for the BA0.5 specimen are presented in Figure 12. Figure 12c,d show the weights of Ca (yellow), C (red), O (green), Si (blue), and B (purple) as 0.13%, 52.60%, 32.95%, 3.47%, and 10.85%, respectively. 

Figure 13 presents the BA1 specimen’s analysis results. By weight, the components of Ca (yellow), C (red), O (green), Si (blue), and B (purple) are 1.87%, 27.69%, 50.27%, 9.13%, and 11.05%, respectively (Figure 13c,d). 

The analysis results for the BA1.5 specimen are presented in Figure 14. Figure 14c,d show that the weights of Ca (yellow), C (red), O (green), Si (blue), and B (purple) are 1.42%, 48.59%, 32.79%, 5.76%, and 11.44%, respectively.

The distribution of boric acid is seen in the EDS analysis of all BA-added specimens. The increase of BA is seen to affect the increase in BA amounts in the analysis results in Figure 12, Figure 13 and Figure 14. In this study, it was experimentally established that the increase in the BA additive has a positive effect on hardness (Table 1).

### 3.6. Effect on Failure Modes

**Figure 15:**
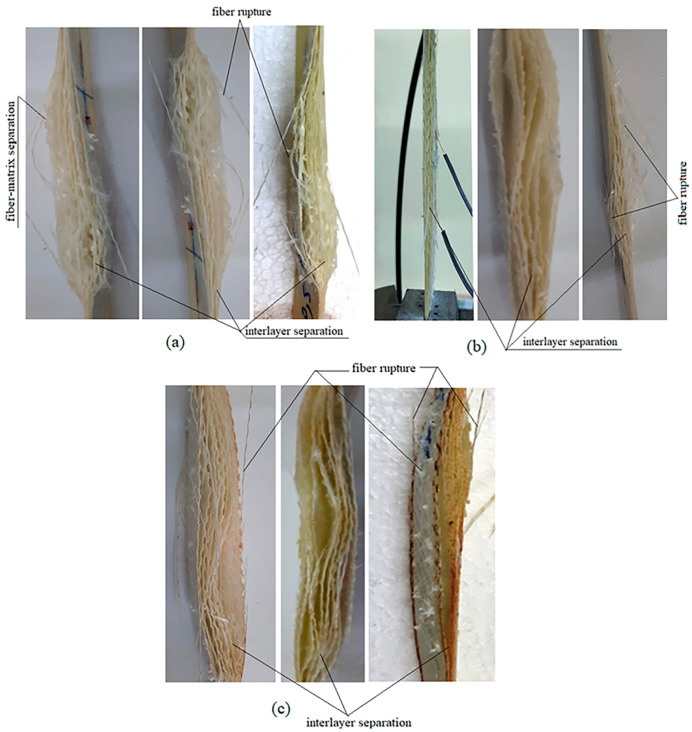
Failure modes: (**a**) BA0.5 specimens, (**b**) BA1 specimens, and (**c**) BA1.5 specimens.

In the tensile test of the specimens, the failure modes obtained from all specimens with BA0.5, BA1, and BA1.5 ratios were observed. It was determined that the layers separated with loading during the test. Figure 15 shows the failure modes of all three specimens subjected to the test.

In the BA0.5 specimens, fiber rupture, fiber-matrix separation, and interlayer separation were observed (Figure 15a). In BA1 and BA1.5 specimens, interlayer separation and fiber rupture were more apparent (Figure 15b,c).

The highest strength values were obtained from the BA0.5 specimen, and it was determined that this value decreased as the BA ratio increased (Table 1). It is thought that the BA additive does not form a good interface, does not create good adhesion, and creates separations between the layers, and this situation causes the failure behaviors in Figure 15. It is also thought that the negative effect on compressive strength is also due to interlayer separation. Bağcı and İmrek stated in their study [26] that, when BA test specimens were examined in terms of deformation, the fibers formed remarkable patterns and caused deeper grooves on the surfaces. Therefore, they stated that BA would not be well compatible with the main matrix and therefore would lead to weak bonding forces [26]. This determination of the authors supports the reason for the failure we obtained in this study.

## 4. Conclusions

The effects of adding 0, 0.5, 1, or 1.5% by weight of boric acid on the thermal and mechanical properties of glass fiber-reinforced epoxy composite plates were examined experimentally in this study. DMA, TGA, and DSC analyses were performed on specimens, and mechanical testing was performed to obtain these properties. BA distribution was observed in the specimens using SEM and EDS analysis. At the same time, the effect of BA on failure modes was investigated. The following results were obtained in the study:

- With the exception of compressive strength, BA improved its mechanical properties. The highest values were obtained from the BA0.5 specimen.
- As the BA ratio increased, the hardness value increased.
- Tg obtained with the help of DMA and DSC analyses have been found to be compatible. The addition of BA decreased the Tg.
- The TGA analysis revealed that, when the BA ratio increased, weight loss decreased.
- The lowest value for both E′ and E″ was obtained from specimen BA1.
- The E′ value obtained from the BA0.5 specimen was 22.65% higher than that obtained from the BA0 specimen.
- The E″ value obtained from the BA0 specimen was 11.28%, 74.36%, and 49.46% higher than the values obtained from the BA0.5, BA1, and BA1.5 specimens, respectively.
- The highest and lowest tan δ values were obtained from BA0 and BA1.5 specimens, respectively. The value obtained from specimen BA0 was 102.14% higher than the value obtained from specimen BA1.5.
- All specimens with the addition of BA had fiber/matrix failures and interlayer separation, distinctly.
- This study can be improved by using BA in different ratios in different fabrics and resins. Also, the effect of BA on peeling and impact tests can be investigated.

## Figures and Tables

**Table 1:** Obtained mechanical properties.

Boric Acid (BA)	Mechanical Properties						
	Tensile Stress (MPa)-(X_t_)	Compressive Stress (MPa)-(X_c_)	Shear Stress (MPa)-(S_12_)	Modulus of Elasticity (MPa)-(E_12_)	Modulus of Shear (MPa)-(G_12_)	Poissons’s Ratio-ʋ_12_	Hardness (HV0.3/10)
0% SD	448.38 [41] 2.53	292.87 [41] 2.31	119.81 [41] 3.57	22,085.98 [41] 5.78	4087.78 [41] 6.57	0.16 [41] 0.01	31 [41] 0.40
0.5% SD	559.49 2.32	246.92 2.64	130.29 1.06	27,636.93 4.51	4547.18 4.04	0.18 0.01	35.2 0.76
1% SD	463.99 2.19	151.91 1.47	124.22 1.53	26,538.28 2.08	4211.93 2.65	0.17 0.01	38.0 0.58
1.5% SD	463.49 2.05	195.46 2.80	126.95 1.25	25,320.46 2.65	4379.17 1.53	0.18 0.005	52.9 0.25

**Table 2:** Results of the specimens’ TGA analysis.

Specimen Type	Start (°C)	End (°C)	Weightloss (%)
BA0	293.49 [41]	490.80 [41]	20.512 [41]
BA0.5%	302.89	490.80	15.745
BA1%	302.20	490.77	14.732
BA1.5%	302.79	490.40	14.212

## Data Availability

The original contributions presented in the study are included in the article. Further inquiries can be directed to the corresponding author.
